# Water Dressings

**Published:** 1874-11

**Authors:** 


### WATER DRESSINGS,

Cold or tepid, have an almost marvellous effect in curing cuts and wounds and bruises. Thus, bring the sides of the wound together, wrap a rag around so as to keep them so, then keep them constantly wet, not disturbing them until the cure is complete, which is effected by keeping down the inflammation. Cuts, bruises and wounds bound up in a rag, dipped in warm liquid glue, are speedily cured without any other treatment; renew the dressing every three or four days. It is a protection against additional injury, and perfectly excludes the air and dust.

